# Brazilian network for the surveillance of maternal potentially life threatening morbidity and maternal near-miss and a multidimensional evaluation of their long term consequences

**DOI:** 10.1186/1742-4755-6-15

**Published:** 2009-09-24

**Authors:** Jose G Cecatti, João P Souza, Mary A Parpinelli, Samira M Haddad, Rodrigo S Camargo, Rodolfo C Pacagnella, Carla Silveira, Dulce T Zanardi, Maria L Costa, João L Pinto e Silva, Renato Passini, Fernanda G Surita, Maria H Sousa, Iracema MP Calderon, Lale Say, Robert C Pattinson

**Affiliations:** 1 Department of Obstetrics and Gynecology, School of Medical Sciences, University of Campinas, Brazil; 2 UNDP/UNFPA/WHO/World Bank Special Programme of Research, Development and Research Training in Human Reproduction, Department of Reproductive Health and Research, World Health Organization, Geneva, Switzerland; 3 CEMICAMP - Campinas Center for Studies in Reproductive Health, Campinas, Brazil; 4 Department of Gynaecology and Obstetrics, Botucatu Medical School, São Paulo State University, Brazil; 5 Obstetrics and Gynaecology Department, University of Pretoria, South Africa

## Abstract

**Background:**

It has been suggested that the study of women who survive life-threatening complications related to pregnancy (maternal near-miss cases) may represent a practical alternative to surveillance of maternal morbidity/mortality since the number of cases is higher and the woman herself is able to provide information on the difficulties she faced and the long-term repercussions of the event. These repercussions, which may include sexual dysfunction, postpartum depression and posttraumatic stress disorder, may persist for prolonged periods of time, affecting women's quality of life and resulting in adverse effects to them and their babies.

**Objective:**

The aims of the present study are to create a nationwide network of scientific cooperation to carry out surveillance and estimate the frequency of maternal near-miss cases, to perform a multicenter investigation into the quality of care for women with severe complications of pregnancy, and to carry out a multidimensional evaluation of these women up to six months.

**Methods/Design:**

This project has two components: a multicenter, cross-sectional study to be implemented in 27 referral obstetric units in different geographical regions of Brazil, and a concurrent cohort study of multidimensional analysis. Over 12 months, investigators will perform prospective surveillance to identify all maternal complications. The population of the cross-sectional component will consist of all women surviving potentially life-threatening conditions (severe maternal complications) or life-threatening conditions (the maternal near miss criteria) and maternal deaths according to the new WHO definition and criteria. Data analysis will be performed in case subgroups according to the moment of occurrence and determining cause. Frequencies of near-miss and other severe maternal morbidity and the association between organ dysfunction and maternal death will be estimated. A proportion of cases identified in the cross-sectional study will comprise the cohort of women for the multidimensional analysis. Various aspects of the lives of women surviving severe maternal complications will be evaluated 3 and 6 months after the event and compared to a group of women who suffered no severe complications in pregnancy. Previously validated questionnaires will be used in the interviews to assess reproductive function, posttraumatic stress, functional capacity, quality of life, sexual function, postpartum depression and infant development.

## Background

Currently, more than half a million maternal deaths occur annually worldwide. Although an extremely rare event in developed countries, maternal mortality is higher in less developed countries. Better social conditions, better medical care in cases of severe complication and family planning are factors that contribute to reducing maternal mortality [1].

Nevertheless, quantifying maternal mortality in Brazil is a complex task. The Ministry of Health estimates the maternal death ratio at 75 maternal deaths per 100,000 liveborn infants [2]. Reflecting the complexity of this estimate, other agencies, using different methods, have calculated maternal death ratios twice or even four times higher than the official figures [3,4].

Notwithstanding, the recorded cases of maternal deaths constitute a tiny proportion of the whole problem. Around the world, millions of women present severe maternal complications every year and the precise size of this specific population currently remains unknown. For this reason, women who have survived severe complications of pregnancy have in recent years sparked the attention of investigators and healthcare administrators. The World Health Organization (WHO) developed the maternal near-miss approach, a tool to uniformly identify near-miss cases and evaluate quality of care provided to women presenting severe complications. WHO defines a maternal near miss case as a woman who nearly died but survived a complication that occurred during pregnancy, childbirth or within 42 days of termination of pregnancy [5].

Therefore, the study of maternal near-miss cases has been suggested as a practical alternative to the surveillance of maternal morbidity and mortality, mainly in view of the larger number of cases and because the woman herself is able to provide information on the event and on the difficulties she had to face. It is believed that auditing near-miss cases would enable even smaller services to evaluate how the determinants of severe maternal morbidity (and consequently the determinants of maternal death) affect their users and services [6,7].

In addition, little is known on the long-term repercussions of severe, life-threatening complications related to pregnancy. An acute stress disorder associated with the occurrence of severe maternal complications has been suggested, but further research is needed. [8]. The repercussions of these events may lead to adverse effects in the women and their children, may negatively affect their quality of life and may persist for extended periods of time after the event [9-12].

Among the possible repercussions, studies have been carried out to evaluate the psychological impact and occurrence of posttraumatic stress disorder (PTSD), postpartum depression and changes in sexual health following delivery [10,13-17]. Considering that other factors such as mode of delivery, medical interventions and obstetrical complications [9,18,19] negatively affect women's quality of life, it is probable that in dramatic situations such as near-misses such repercussions would be even more evident. According to some authors, evaluation of the state of health, quality of life and sexual function of patients who suffered severe complications is poorer in the immediate postpartum period [15,20-23].

Nevertheless, doubts remain with respect to the long-term health status of women who suffer severe acute maternal morbidity and near-miss. Investigation of various aspects related to mental health and quality of life may offer a valuable perspective on the effect of maternal morbidity on the life of these women.

Studying the occurrence of severe complications in pregnancy and the factors associated with this event will result in a greater understanding of the process that occurs in these women taking them from a state of health to one of sickness. Further knowledge on this issue may collaborate towards improving public policies and the healthcare provided to women who develop severe acute maternal morbidity.

Therefore, the objective of the present project is to evaluate this issue using clear goals to differentiate it from previous studies. These goals include estimating the frequency of the occurrence of maternal near-miss using a uniform set of criteria, carrying out a multicenter investigation into the quality of care provided to women with severe complications of pregnancy and performing a longitudinal evaluation of the quality of life of these women following the event.

## Objectives and Hypothesis

The overall objective is to develop a nationwide network of scientific cooperation for the surveillance of severe maternal complications and maternal near-miss and their consequences.

### Specific objectives

• To determine the frequency of maternal near-miss in healthcare facilities of different levels of complexity situated in different regions of Brazil, using the World Health Organization (WHO)'s new set of criteria for near-miss [5];

• To determine the frequency of non-near-miss severe maternal morbidity in these facilities using specifically defined potentially life threatening conditions;

• To evaluate the association between the indicators of organ dysfunction used to define maternal near-miss and the risk of maternal death;

• To determine the frequency of near-miss and non-near-miss severe maternal morbidity according to age-group and specific causes;

• To examine the occurrence of avoidable factors and other factors associated with maternal near-miss;

• To investigate the repercussions of severe maternal morbidity and near-miss on the quality of life of survivors up to six months after the event;

• To investigate the presence of sexual dysfunction, posttraumatic stress disorder and postpartum depression, as well as women's perception of their functional status in routine activities in the six months following an occurrence of severe maternal morbidity.

• To investigate the immediate perinatal outcome and subsequent neuromotor and weight-height development in children born from pregnancies associated with severe maternal morbidity.

### Main hypotheses

In survivors of severe acute maternal morbidity:

• health and quality of life would be poorer;

• posttraumatic stress would be more common;

• postpartum depression would be more common;

• sexual function would have deteriorated and the woman's return to sexual activity would take longer;

• functional status in routine activities would be evaluated as poorer.

In the children born from a pregnancy associated with severe maternal morbidity:

• immediate perinatal outcome would be poorer;

• the occurrence of impaired neuromotor and weight-height development would be significantly higher.

## Methods/Design

This study has two components: a multicenter cross-sectional study and a concurrent cohort study.

The cross-sectional study will be implemented in 27 referral obstetric units in different geographical regions of Brazil, which have already joined the initiative for building a national network for studies on maternal and reproductive health. Over a 12-month period, the principal and local investigators will carry out prospective surveillance and will collect data for the identification of maternal near-miss and non-near-miss cases, severe maternal morbidity (potentially life threatening conditions) and maternal deaths. To determine the number of collaborating centers to be included in the present study, calculation of sample size took into consideration the number of deliveries that would have to be monitored to identify cases of near-miss and maternal deaths. Previous studies have estimated a maternal near miss incidence of approximately 8 cases per 1000 deliveries [24] and a Brazilian maternal mortality ratio of 140 per 100,000 LB. Therefore, a total of approximately 75,000 deliveries would have to be monitored in order to identify around 100 maternal deaths and 600 maternal near miss cases. These numbers are believed to be sufficient to evaluate the use of the new criteria for near-miss established by the World Health Organization in 2009 [5] and to perform analysis allowing for level of complexity of health facility, age group and specific cause.

The study population will consist of all the women admitted to the participating hospitals during the study period in whom organ dysfunction is registered (maternal near-miss, Appendix 1), in whom one of the diagnoses defined as non-near-miss severe maternal morbidity is present (Appendix 2), and those who died or were transferred to another healthcare service because of their bad health condition.

For the multidimensional analysis of the repercussions of severe maternal morbidity, a concurrent cohort, specific population study will be carried out with an externally selected comparison group. The main exposure factor will be the occurrence of severe maternal morbidity (both maternal potentially life threatening or near miss conditions). During the second half of the cross-sectional study, a sample of women identified as having severe maternal morbidity will be selected and invited to participate in the longitudinal evaluation. There will be a comparison group composed of women who did not suffer severe maternal morbidity. These women will be randomly selected externally in a proportion of 1:1 from postpartum women in the rooming-in wards of the same maternity hospitals as the cases. Controls will be selected at random and balanced according to mode of delivery, maternal age and gestational age at the time of delivery.

### Main outcomes

#### Maternal near-miss

A woman who fulfills one of the clinical, laboratory or management criteria representing severity as defined by WHO [5] and who survives a complication occurring during pregnancy, childbirth or within 42 days postpartum.

#### Maternal potentially life threatening condition

A condition of severe morbidity found in women during pregnancy, childbirth or in the puerperium, classified as potentially life threatening conditions [5], including hemorrhagic or hypertensive disorders, other systemic disorders, and indicators of severe management (Appendix 2).

#### Main cause of complication/death

classification of the determinant main cause of the complication identified among cases and/or the main cause of death.

#### Maternal death

Death of a woman during pregnancy or within a 42-day period following the end of pregnancy irrespective of the duration or localization of the pregnancy, resulting from any cause related to or aggravated by the pregnancy or by measures taken with respect to it; however, not from accidental or incidental causes.

#### Conditions at birth

Vital status of the newborn infant as recorded on the medical chart, dichotomized into live or intrauterine death.

#### Vitality of the newborn infant

Evaluation of the newborn infant according to 1^st ^and 5^th ^minute Apgar scores as shown on the medical chart, classified from 0 to 10.

#### Neonatal outcome

Condition of the newborn infant at the time of data collection, identified from a review of the medical charts and classified as: discharged from hospital together with the mother, early neonatal death (<7 days) or late neonatal death (7-28 days).

#### Quality of life

The woman's perception of her position in life within the cultural context and value system in which she lives and in relation to her goals, expectations, health, standards and concerns (WHO); identified by the investigators using a standard SF-36 form.

#### Posttraumatic stress

Symptoms of intrusion, avoidance and hyperarousal following the occurrence of a pregnancy with severe complications; identified by the investigator using a standard questionnaire (PTSD - Checklist CV).

#### Ideal number of children

Number of children that the woman considered ideal prior to and following the index pregnancy.

#### Return to sexual activity

Time taken by the woman to recommence sexual activity after delivery and reason given for not recommencing sexual activity.

#### Sexual function

Sexual function and response; identified by the investigator using a standard questionnaire (*Female Sexual Function Index - FSFI*).

#### Postpartum depression

Depressive symptoms following the occurrence of a pregnancy with severe complications; identified by the investigator using a standard questionnaire (Edinburgh Postnatal Depression Scale - EPDS).

#### Functional status

Perception of the woman with respect to her functional status in six items related to her routine activities (understanding and communicating, getting around, self-care, getting along with people, life activities in the home/at work and participation in society), classified from 0 to 100 (from best to worst) [25].

#### Neuromotor development in the child born from the index pregnancy

Process of changes in motor behavior that involve both maturation of the central nervous system and interaction with the environment and stimuli given during the child's development; identified by the investigator using the Denver II - Revised Denver Developmental Screening Test [26].

#### Weight-height development of the child born from the index pregnancy

Process of weight and height increment during the child's development, weight measured in grams and height in centimeters, using scales and anthropometer, classified as adequate or inadequate for age, according to the standards of the World Health Organization [27].

#### Control variables

maternal age, marital status, place of residence, number of previous pregnancies, parity, previous abortions, previous Cesarean sections, number of children, mode of delivery, gestational age, birthweight, gender of neonate, condition of neonate at discharge, condition of mother at discharge.

### Data Collection and Procedures

#### Cross-sectional component

Research assistants, referred to as local coordinators, will review the charts of hospitalized patients on a daily basis in search of cases with one of the conditions identifying severity (Appendix 2). In cases found with these diagnoses, the relevant hospital records will be reviewed for data collection following the women's hospital discharge, death or transfer to another healthcare facility. Data unavailable on the chart but of interest to the study will be obtained from the attending medical team. For each case included, data will be collected on the demographic and obstetric characteristics of the patient, the primary determinant of maternal near-miss (the first complication to occur in the chain of events leading to severe maternal morbidity), the duration of hospitalization (prior to delivery, following delivery and total time), the occurrence of indicators of maternal near-miss at any time during hospitalization, indicators of perinatal outcome and conditions of the woman at discharge from hospital.

These data will be collected on a previously coded form developed specifically for this purpose. A central database will be constructed and the data will be included by the local investigators themselves using electronic forms. The manually completed forms will be filed and made available at technical visits for the purpose of quality control.

For the electronic inclusion of data, each center will have its own restricted area on the study website where password-protected access will be granted only to cases included at that center. An overview of all the cases included in the network will be provided in the form of monthly graphs and tables containing the number of cases included by each center. In addition, the reported diagnoses will be provided by the coordinating center on the main page of the website.

In cases of near-miss, data will be collected on avoidable factors responsible for their occurrence (delays). These factors will be classified into those related to infrastructure, the patient or the healthcare professionals. Avoidable factors related to infrastructure include cases in which difficulties in obtaining supplies or medication, transportation, communication, blood components or monitoring and treatment may have led to less than ideal care. Factors related to the patient include those generated by the patient herself or her family, either by delaying seeking professional care or by refusing treatment. Factors related to the healthcare team include delays in defining the correct diagnosis and/or inappropriate management.

The degree of complexity at each hospital will be evaluated using an adapted version of the hospital complexity index developed for the WHO Global Survey project [28]. Participating institutions will provide information on a monthly basis via the website on the total number of deliveries, live births and maternal deaths that occurred the previous month. These data will be confirmed by the principal local investigator after data collection is finished.

To minimize the number of uncertainties that research assistants may face during data collection, a manual of operation was produced containing all the necessary information on how to use the internet, how to complete the written and electronic forms and how to access the database of each individual center, as well as information regarding the standardization of diagnostic definitions.

A meeting will be held with the investigators and local coordinators of each center (two individuals from each center) at the study coordinating center immediately preceding initiation of data collection in order to provide adequate training and clarify any queries regarding the data collection process and use of the website. Sometime after the initiation of data collection, a meeting of the study's Steering Committee will also be held. A second meeting will take place involving only the local investigators after data collection has finished to discuss facts related to the previous process, disclosure of partial results, scheduling of the preliminary and final analyses, agreement on papers to be written on the results and assignment of responsibility regarding execution of each item in this process.

#### Longitudinal component

As in the cross-sectional component, women with one of the conditions indicative of severity will be selected as potential subjects for longitudinal evaluation. Once identified, research assistants who are not involved in the cross-sectional portion of the study will invite eligible women to participate in the longitudinal evaluation of the study. Women who agree to take part will be asked to sign an informed consent form and two CATI (computer assisted telephone interview) will be scheduled for 3 and 6 months postpartum plus a medical visit with the woman and the newborn infant six months following delivery.

For the control group, all women admitted to the hospital for obstetric care in the same facility on the same day on which a case has been identified and who have none of the conditions indicating severity will be eligible. Following a process of randomized selection balanced according to mode of delivery, maternal age and gestational age at the time of delivery, women in the control group will be invited to participate in the study by the research assistants in the same way as candidates to the study group. Three months after delivery, the study call center will contact the women to carry out the first step in data collection. At the time of this contact, the interviewers will again go over the objectives of the study and will apply standard questionnaires designed to investigate quality of life and postpartum depression. This interview is estimated to last around 20 minutes.

At six months postpartum, the study call center will contact the women again to carry out the second step in data collection. At this contact, the interviewers will go over the study objectives once again and apply the same standard questionnaires on quality of life and postpartum depression, lasting no more than 20 minutes. In the case of women who do not have a telephone, a reminder letter will be sent asking them to phone the study call center at the sixth month postpartum to enable the interview to take place.

At the end of the 6-month telephone interview, the interviewer will confirm the date, time and place of the visit that was previously scheduled when the women were still in hospital. The women will be reminded that they should bring the baby to the visit. Even if they do not authorize the participation of their infants in the study, the women will be invited to return to the hospital and answer the questionnaires. The interview will be carried out by a trained female interviewer, who will apply standard questionnaires to evaluate posttraumatic stress disorder, sexual function and the woman's perception of her functional status in routine activities, taking no more than 35 minutes for each woman. After the mothers have answered the questionnaires, the weight, height and neuro-psychomotor development of the infants will be evaluated by a specially trained pediatrician, taking around 20 minutes. Finally, the women will receive a token cash payment as a contribution towards their transportation and food costs while attending this visit.

The following instruments will be used for data collection:

#### Posttraumatic Stress Disorder (PTSD) Checklist - Civilian Version (PCL-C)

This questionnaire has been validated in Brazil to screen for the diagnosis of posttraumatic stress disorder. It contains 17 items in which women will indicate to what extent she has been disturbed by symptoms over the past month on a scale of 1-5 (ranging from not at all to a lot). A score ≥ 3 (a medium score) for any one of the items is considered indicative of a clinically significant symptom.

#### Medical Outcomes Study 36-Item Short-Form Health Survey (SF36)

This is a generic questionnaire for evaluating quality of life that has been validated for use in Brazil. It is multidimensional with 36 items in 8 scales: physical functioning, role-physical, body pain, general health, vitality, social functioning, role-emotional and mental health. Final scores vary from 0 to 100 (poorest to best).

#### Female Sexual Function Index

A multidimensional questionnaire used to evaluate female sexual function consisting of 19 questions in 6 domains: desire, arousal, lubrication, orgasm, satisfaction and pain. Final scores vary from 2 to 36, a cut-off point < 26 having been proposed as determinant of sexual dysfunction. This questionnaire has been culturally adapted for use in Brazil.

#### Edinburgh Postnatal Depression Scale (EPDS)

A questionnaire used to screen for symptoms of depression and anxiety in the postpartum period, containing 10 questions that may be self-administered. A final score ≥ 10 has been defined as the cut-off point of greatest sensitivity in screening. The tool has been validated for use in Brazil.

#### The World Health Organization Disability Assessment Schedule II (WHODAS II)

A 36-item questionnaire used to evaluate the individual's perception of herself and her functional status, consisting of six activity domains related to the woman's routine activities (understanding and communicating, getting around, self-care, getting along with people, life activities in the home/at work and participation in society), on a 6-level scale varying from (1) no difficulty to (6) extreme difficulty/cannot do. Final score varies from 0 to 100 (from best to worst) [25].

#### Neuro-psychomotor development of the child

The Denver Developmental Screening Test II consists of 125 tasks or items organized in the form of tests of 4 general functions: personal-social, fine motor-adaptive, language and gross motor. At the end, a behavior test is applied that helps the examiner subjectively observe the overall behavior of the child and obtain an impression on how the child uses his/her skills.

### Quality control

Quality control procedures will be adopted and include techniques such as reviewing completed forms, checking data entry, repeating data collection for selected medical charts and the use of a detailed manual of operation. Initial quality control of data collection will be performed by the local investigator prior to and during electronic data entry of the forms in order to identify any possible inconsistencies in the data.

A second quality control procedure will be carried out by one of the principal investigators, who will visit the participating centers. At this visit, consistency will be verified between the manual records on file and the data contained in the electronic forms. In addition, a random evaluation will be made of hospital records.

For the quality control of the longitudinal component, 10% of the records at each participating center will be randomly selected at the end of individual data collection and contact will once again be made with the patient in order to verify the data obtained at the first interview. The local investigators will maintain a record of any problems occurring during the study and any queries will be raised with the country coordinator of the project.

### Data analysis

Data analysis will be performed in sub-groups according to the time of occurrence of the near-miss or severe maternal morbidity (in adolescence, older ages or at another time in the woman's reproductive life) and determining cause (hypertension, hemorrhage, abortion or other causes). The rates of maternal near-miss will be calculated for each collaborating center using the WHO maternal near miss approach [5], and frequencies of non-near-miss severe maternal morbidity will be calculated using specific defined diagnoses. General estimates will be calculated together with their respective 95% confidence intervals. The association between organ dysfunction and maternal death will be estimated using odds ratios, likelihood ratio test and their respective 95% confidence intervals. In addition, relative risks will be calculated for sexual dysfunction, postpartum depression, posttraumatic stress disorder, deterioration in quality of life, deterioration in the woman's perception of her own functional status in routine activities, risk of adverse perinatal outcome and risk of impaired neuromotor and weight-height development in the children born from the pregnancy associated with severe maternal morbidity.

### Results obtained from the preliminary project

Initially, a meeting was held during the Brazilian national congress of Gynecology and Obstetrics in November, 2007, and attended by representatives of 35 healthcare facilities in Brazil. At this meeting, the main points featured in the initial concept of the project were presented and an invitation was made to institutions interested in participating in a Brazilian network on the topic. Those who were interested in participating filled out a registration form with the addresses and characteristics of their respective healthcare institutions. In December 2007, an electronic form was sent to them to be completed with specific information. In accordance with the data received, 27 of these candidate healthcare institutions were selected to participate in the network, taking regional characteristics, geographic distribution, level of complexity and the number of deliveries performed into consideration.

In August 2008, a meeting with representatives from all the centers was held at the coordinating center in Campinas. At this meeting, the proposal was presented and discussed in detail, and suggestions were incorporated into the final version of the protocol. Participating center representatives were identified, the operational issues involved in implementing the study and the theoretical concepts were discussed, and the final version of the research project was defined. Concurrently, a signed commitment was undertaken by each representative to participate in the Brazilian Network for the Surveillance of Severe Maternal Morbidity: the Brazilian Network of Studies in Reproductive and Perinatal Health was created. A Steering Committee was also designated for the study.

### Ethical aspects

The coordinating center has already obtained the approval of the local Institutional Review Board and of the National Council for Ethics in Research (CONEP) of the Brazilian Ministry of Health for both components of the project. The participation of the collaborating centers in this study will only be confirmed after the project has been approved by their respective Institutional Review Boards. Individual signed informed consent will not be requested from the women involved in the cross-sectional analysis, since this study does not involve any type of intervention that could adversely affect their treatment; the data of interest will be obtained retrospectively from the patient's charts and without identifying the woman. Therefore, a waiver of the requirement for signed informed consent was obtained. It is understood that there is no other way of obtaining concrete, reliable information on cases of severe maternal morbidity or death, since these patients are unable to give their consent. However, informed consent will be obtained from the women involved in the longitudinal component of the study. All the principles regulating research in human beings will be respected.

Based on the questionnaires applied, women diagnosed with some type of pathological condition, who are not receiving medical care, will be referred to healthcare facilities equipped to provide them with follow-up care. Women who have already received a diagnosis of a pathological condition but are not being followed up by a physician will also be referred to an appropriate healthcare service.

### Technical and scientific contributions expected from the project

Brazil is a country with very high proportion of births taking place in health facilities (around 97%). The results of the present study will permit a prospective evaluation of severe maternal morbidity and deaths nationwide through the participation of healthcare facilities with different regional characteristics. No multicenter collaborative studies of this dimension are currently being carried out in healthcare institutions in Brazil in the field of Reproductive Health, and no data thus obtained are currently available. In addition to the specific study of maternal health hazards, the organizational structure required by this project will guarantee continuity of the investigation into various conditions of interest to public health beyond the period in which this study will be conducted. The implementation of a collaborative network is essential for expanding the production of substantive research in the field of maternal and perinatal health in Brazil.

Certainly, the availability of resources for the implementation and development of the Brazilian Network for the Surveillance of Severe Maternal Morbidity will lead to new scientific findings relevant to Brazil and other countries. Concomitantly, this will permit the construction of an innovative technological base from which health data may be obtained on a continuous basis, providing the evidence required to institute a real and effective improvement in the quality of life and health of the population. This network is committed to participating in future collaborative studies in the areas of perinatal and women's healthcare. The implementation of a series of multicenter studies is anticipated in this area in a way never before achieved in this country. This fact gives greater power to the results, which will therefore be more representative of the country, a particularly interesting achievement bearing in mind the wide ethnic, cultural and social diversity of the Brazilian population.

We hope that this initiative contributes to the improvement of health care and for the reduction of maternal and perinatal morbidity and mortality.

## Competing interests

The authors declare that they have no competing interests.

## Authors' contributions

The idea for the study arose in a group discussion with all authors. The first version of the protocol was drafted by JPS and JGC, then complemented with the suggestions of the others. RCP and RSC were responsible for including the initial proposal for a multidimensional evaluation of consequences. SMH was responsible for the final, complete version of the protocol. JGC supervised the whole process. All authors contributed to the development of the study protocol and approved the final version of the manuscript.

## Appendix 1: Criteria defining Near-Miss (WHO)*

A woman who fulfills one of the following criteria and survives a complication during pregnancy, childbirth or in the 42 days postpartum should be considered a near-miss.

### Clinical Criteria

Acute cyanosis

Breathing rate > 40 or < 6

Oliguria unresponsive to fluids or diuretics

Loss of consciousness for ≥ 12 hours

Unconscious, no pulse/heartbeat

Jaundice concomitantly with preeclampsia

Gasping

Shock

Coagulation disorders

Cerebrovascular accident

Total paralysis

### Laboratory Criteria

Oxygen saturation <90% for > 60 minutes

Acute thrombocytopenia (<50,000 platelets)

Creatinine ≥ 300 μmol/l or ≥ 3.5 mg/dL

Bilirubin >100 μmol/l or > 6.0 mg/dL

Unconscious, presence of glucose and ketoacidosis in urine.

Lactate > 5PaO2/FiO2 < 200

pH < 7.1

### Management Criteria

Use of continuous vasoactive drug

Dialysis for treatment of acute kidney failure

Puerperal hysterectomy due to infection or hemorrhage

Cardiopulmonary resuscitation (CPR)

Transfusion ≥ 5 units of red blood cell concentrate

Intubation and ventilation for a period ≥ 60 minutes, unrelated to anesthesia*

Modified from [5]

## Appendix 2: Indicators of non-near-miss severe maternal morbidity (potentially life-threatening conditions) *

### Hemorrhagic disorders

Abruptio placentae

Placenta accreta/increta/percreta

Ectopic pregnancy

Antepartum hemorrhage

Postpartum hemorrhage

Ruptured uterus

Abortion with severe hemorrhage

### Hypertensive disorders

Severe Preeclampsia

Eclampsia

Severe hypertension

Hypertensive encephalopathy

HELLP syndrome

### Other systemic disorders

Endometritis

Pulmonary edema

Respiratory failure

Seizures

Sepsis

Thrombocytopenia <100,000

Thyroid crisis

### Management indicators of severity

Blood transfusion

Central venous access

Hysterectomy

ICU admission

Prolonged hospital stay (>7 postpartum days)

Intubation not related to anaesthetic procedure

Return to operating room

Major surgical intervention

*Modified from [5]
